# Clinico-epidemiological characteristics of patients with vitiligo in Bangladesh

**DOI:** 10.1016/j.jdin.2023.06.012

**Published:** 2023-07-06

**Authors:** Md. Rashidul Hasan, Gulam Kazem Ali Ahmad, Md. Razu Ahmed, Md. Tauhidur Rahman, Afsana Nahid, Salwa Islam, Anindita Das Barshan, Mohammad Jahid Hasan

**Affiliations:** a Department of Skin and Venereal Diseases, US Bangla Medical College Hospital, Dhaka, Bangladesh; b Department of Skin and Venereal Diseases, Shah Mohiuddin Medical College Hospital, Rajshahi, Bangladesh; c Rangpur Medical College, Rangpur City, Bangladesh; d Director General of Health Services, Dhaka, Bangladesh; e Department of Dermatology and Venereology, Dhaka Medical College Hospital, Dhaka, Bangladesh; f Pi Research and Development Center, Dhaka, Bangladesh; g Department of Medicine, Dhaka Medical College Hospital, Dhaka, Bangladesh

**Keywords:** autoimmune, Bangladesh, clinical patterns, disease, epidemiology, vitiligo

*To the Editor:* Vitiligo is an acquired idiopathic skin disorder characterized by pigmentation of the skin and mucous membrane. Skin involvement might range from small pale or white macules to near-total depigmentation of the body. In a developing country such as Bangladesh, there is a slew of skin disease cases, and among these, vitiligo is a distinctive one.^1^ Patients with vitiligo often experience discrimination and stigma in Bangladesh. Due to the belief that it is contagious, people avoid contact, further isolating them. Moreover, it was reported that majority of them suffer from marital problems because vitiligo evokes misconceptions and prejudice within relationships. This deprivation of mental and physical support often leads to emotional distress, depression, and worsening of the quality of life of patients with vitiligo.^2^ Information regarding vitiligo is limited, and there are no recognized epidemiological data on the prevalence and incidence of vitiligo in Bangladesh; therefore, this cross-sectional study aimed to provide insight into the clinico-epidemiological characteristics of patients with vitiligo in Bangladesh.

A total of 539 vitiligo cases confirmed by detailed history, clinical findings, and Wood’s lamp test were included, of which 53.1% were females, and the mean age of onset was 4.8 years. On query, 20% provided a family history of vitiligo, and 4.6% had autoimmune diseases, of whom 3.7% had thyroid disease. The common sites of vitiligo are the face and limbs. Of all the cases, 89.6% had nonsegmental vitiligo, and leukotrichia and Koebner phenomenon were reported by 17% and 33.2% of the patients with vitiligo, respectively. Triggering factors, such as psychological stress and exposure to chemicals, were reported by none of the patients, and physical trauma was reported by 21.3%. Moreover, 78.8% of the patients had their vitiligo in progressive mode, 51.4% of respondents did not seek any treatment, and 44.9% of them relied on both topical and systemic approaches of treatment for vitiligo. None of the patients underwent epidermal grafting and surgical treatments (Tables I and II).

**Table I:** Demographic profile of the respondents

Demographic characteristics	*n*	%
**Sex**∗		
Male	253	46.9
Female	286	53.1
**Age group** (y)∗		
1-10	124	23
11-20	147	27.3
21-30	126	23.4
31-40	65	12.2
41-50	52	9.6
51-60	20	3.7
>60	5	0.9
**Age** (y), mean ± SD	23.22 ± 1.53	
**Residence**†		
Rural	242	45
Urban	291	55
**Occupation**‡		
Government employee	13	2.5
Nongovernment employee	90	17.2
Business	15	2.9
Farmer	11	2.1
Housewife	111	21.2
Unemployed	14	2.7
Others	269	51.4
**Education status**§		
No scholarship	55	11.7
Below SSC	169	35.8
SSC	66	14
HSC	86	18.2
Graduate and above	96	20.3
**Family income**‖		
10,000-20,000	67	15.9
20,000-40,000	319	75.8
>40,000	35	8.3
**Smokeless tobacco use**		
Chewer	13	2.4
Nonchewer	526	97.6
**Smoking habit**		
Smoker	12	2.2
Nonsmoker	527	97.8
**Alcoholism**		
Alcoholic	2	0.4
Nonalcoholic	537	99.6
**Family history of vitiligo**		
Yes	108	20
No	431	80
**Presence of parental consanguinity**		
Present	18	3.3
Absent	521	96.7
**Systemic disease**∗¶		
Diabetes mellitus	20	3.7
Thyroid disease	20	3.7
Anemia	0	0
Rheumatoid arthritis	0	0
Hypertension	9	1.7
Others	2	0.4
**Cutaneous disease**∗		
Halo nevus	2	0.4
Plaque psoriasis	1	0.2
Canities	0	0
Alopecia areata	1	0.2
Lichen planus	0	0
Ichthyosis vulgaris	0	0
Other	0	0
**Ocular disorder**		
Present	29	5.4
Absent	510	94.6

*HSC*, Higher secondary school certificate; *SSC*, secondary school certificate.

∗ Frequency and percentages expressed according to *n* = 539.

† Frequency and percentages expressed according to *n* = 533.

‡ Frequency and percentages expressed according to *n* = 523.

§ Frequency and percentages expressed according to *n* = 472.

‖ Frequency and percentages expressed according to *n* = 421.

¶ Multiple responses considered.

**Table II:** Clinical characteristics of the patients with vitiligo (*n* = 539)

Clinical characteristics	*n*	%
**Site of vitiligo during diagnosis**∗		
Face	253	46.9
Scalp	66	12.1
Neck	68	12.6
Buccal mucosa	13	2.4
Upper limb	194	36
Lower limb	225	41.7
Trunk	126	23.4
Groin/genital	40	7.4
Others	2	0.4
**Age of onset** (y)		
Mean ± SD	4.8 ± 6.90	
**Types of vitiligo**		
Nonsegmental	483	89.6
Segmental	38	7.05
Mixed	0	0
Unclassified	18	3.3
**Presence of other features**		
Leukotrichia	91	17
Koebner phenomenon	179	33.2
**Triggering factors**		
Physical trauma	115	21.3
Psychological stress	0	0
Pregnancy	2	0.4
Drugs	3	0.6
Exposure to sun	6	1.1
Exposure to chemicals	0	0
Others	113	21.0
**Disease stability**		
Progressive	425	78.8
Regressive	13	2.4
Stable	101	18.7
**Treatment approach**		
Topical approach	18	3.3
Systemic approach	2	0.4
Both topical and systemic approaches	242	44.9
Epidermal grafting and surgical treatments	0	0
No treatment	277	51.4
**Types of treatment**∗		
***Topical treatment***		
Tropical steroids	80	14.8
Topical calcineurin inhibitors	243	45.1
Vitamin D analogs	6	1.1
UV therapy	1	0.2
Psoralen plus ultraviolet A	23	4.3
***Systemic treatment***		
Oral corticosteroids	88	16.3
Immunosuppressive agents	5	0.9
Oral antioxidants	196	36.4

∗ Multiple responses were considered.

In our study, a slight female predominance was reported because females tend to be more concerned and stressed regarding cosmetic deformity and, hence, report and seek active treatment more frequently.^2^ The presence of autoimmune disease, early onset of vitiligo, and family history of vitiligo were crucial findings in this study population, similar to other studies that suggested that vitiligo is a complex relationship between genetic loci, earlier age of onset, dysregulation of the immune response, and greater frequency of other autoimmune diseases, particularly in family members of the patients effected by autoimmune diseases.^3^ Moreover, the common sites mentioned were the face and limbs, and it is quite evident that physical trauma is a factor inciting vitiligo because these extremities are more exposed to friction and injuries during daily activities. Despite seeking treatment, most of them reported that their vitiligo was in progressive mode, and over half of the patients did not seek treatment. Limited availability of free noncommunicable disease medicines in public health care facilities, out-of-pocket expenditures for surgeries and phototherapies, and long-term commitment to treatments act as barriers to seeking treatment in Bangladesh.^4^^,^^5^ Our study results suggest that increasing awareness and appropriate counseling to seek treatment for vitiligo are crucial to prevent further progression of the disease in this study population.

## Conflicts of interest

None disclosed.
